# Combined IFN-α and 5-FU treatment as a postoperative adjuvant following surgery for hepatocellular carcinoma with portal venous tumor thrombus

**DOI:** 10.3892/etm.2012.736

**Published:** 2012-10-05

**Authors:** HIROAKI NAGANO, SHOGO KOBAYASHI, SHIGERU MARUBASHI, HIROSHI WADA, HIDETOSHI EGUCHI, MASAHIRO TANEMURA, YOSHITO TOMIMARU, KOJI UMESHITA, YUICHIRO DOKI, MASAKI MORI

**Affiliations:** 1 Departments of Surgery and; 2 Health Science, Graduate School of Medicine, Osaka University, Osaka, Japan

**Keywords:** HCC, PVTT, IFN, chemotherapy, surgery

## Abstract

The efficacy of combination therapy with subcutaneous interferon (IFN)-α and intra-arterial 5-fluorouracil (5-FU) as a postoperative adjuvant for resectable advanced hepatocellular carcinoma (HCC) invading the major branches of the portal vein (PVTT) was examined. The prognosis of HCC with PVTT (Vp3 or 4) is extremely poor. Recently, we reported the possibility of combination therapy with IFN-α and intra-arterial 5-FU for intractable HCC with PVTT as a postoperative adjuvant and this is the second report. Patients with HCC with PVTT were included (n=50). Thirty consecutive patients with HCC and PVTT were treated with 3 cycles of a combination therapy consisting of arterial 5-FU infusion (300 mg/mm^3^/day, 5 days/week, for the initial 2 weeks) and IFN subcutaneous injection (5 MIU, 3 times/week, 4 weeks) as a postoperative adjuvant following hepatic resection; another 20 patients receiving no IFN/5-FU chemotherapy acted as controls. Results for the IFN/5-FU adjuvant treatment group were as follows: disease-free survival (n=9, 15–109 months), survival with recurrence (n=6, 30–92 months), cancer death (n=9, 14–60 months), death from other causes but no recurrence (n=5, 13–87 months) and death from other causes with recurrence (n=1, 22 months). The 1-year survival rate was 100% in patients treated with IFN/5-FU, and 30% in those without IFN/5-FU as historical controls (n=20). There was a significant difference in disease-free and overall survival rates between the two groups (P<0.0001). In conclusion, IFN/5-FU combination therapy may be a very promising postoperative adjuvant treatment for HCC with PVTT.

## Introduction

Hepatocellular carcinoma (HCC) is a common malignancy worldwide and is now the third major cause of cancer-related death in Japan (1). Vascular invasion, particularly portal veinous tumor thrombus (PVTT), is one of the indicators of patient prognosis and has been well documented. The mortality rate is very high in patients with unresectable tumors, and the quality of life (QOL) is poor due to intractable ascites or esophageal bleeding. In such a situation, conventional therapies generally have no clinical effect, therefore, a new strategy is required for patients of advanced HCC with PVTT in the major trunk.

Recently, sorafenib, an oral multikinase inhibitor of the vascular endothelial growth factor receptor, the platelet-derived growth factor receptor and Raf, has been demonstrated to prolong median survival and the time to progression by nearly 3 months in patients with advanced HCC as compared with those administered a placebo (2). However, no complete response and few partial responses (2%) were found in the same study. Although this drug can be used for the treatment of patients with advanced HCC, its clinical effectiveness is still controversial in Japan. According to the consensus-based clinical manual proposed by the Japan Society of Hepatology (3), arterial infusion chemotherapy using an implantable drug delivery system is recommended as one of the treatments for advanced HCC with portal venous invasion. Several recent studies have indicated the beneficial effects of interferon (IFN)-α-based combination chemotherapies for HCC (4–8). We also reported the clinical efficiency of IFN-α and 5-fluorouracil (5-FU) combination therapy for advanced HCC with portal venous tumor thrombi and intrahepatic metastasis (9–11), including the mechanism of the anti-tumor effect (12–19). In addition, we applied this combined chemotherapy (IFN/5-FU) for resectable HCC as a postoperative adjuvant (20) and a multimodal treatment (21).

In the present study, we investigated the clinical effect of IFN/5-FU therapy for resectable advanced HCC with PVTT as a postoperative adjuvant therapy, as an extention of our previous report (20).

## Patients and methods

### Patients

Of the patients with HCC who were admitted and underwent curative hepatic resection at the Department of Surgery, Osaka University Hospital, 50 were included in this study based on the identification of a tumor thrombus either in the major or first branch of the portal vein (Vp3 or 4) (22). Liver function tests and imaging techniques, including computed tomography (CT) with hepatic angiography and arterial portography, revealed that these cases were resectable and subsequently they underwent hepatectomy. Of the 50 patients, 30 patients, from 1998 to 2008, had an intra-arterial catheter inserted through the gastro-duodenal artery with an implanted drug delivery system during the operation to facilitate postoperative adjuvant IFN/5-FU combined chemotherapy (9–11). They were treated with 3 cycles as a postoperative adjuvant. The demographic data of these patients are shown in Table IA. The 15 patients, no. 1–15, are the same patients as in our previous study (20), followed up for a longer period. Another 20 patients, from 1987 to 2007, with the same tumor stage of advanced HCC and Vp3 or 4, underwent surgery but did not receive combined IFN/5-FU therapy. They were treated with appropriate local HCC therapy except for 3 cases (no. 44, 45 and 48) who received only 5-FU intra-arterial chemotherapy without IFN. The demographic data of these patients are shown in Table IB. The 15 patients, no. 31–45, are the same patients as in our previous study (28), followed up for a longer period. The cases, no. 31–43 are the historical controls; no. 44–50 refused IFN/5-FU combined chemotherapy. These 2 groups were compared in terms of features of HCC, hepatic function, surgery, clinical effects, disease-free and overall survival.

The TNM stage and grade of portal vein thrombus were classified according to the 5th edition of the General Rules for the Clinical and Pathological Study of Primary Liver Cancer by the Liver Cancer Study Group of Japan (22). The criteria for selection for intra-arterial combination treatment included i) the absence of extra-hepatic metastases, ii) AST and ALT levels <100 IU/l, iii) a platelet count >80,000/mm^3^, iv) successful implantation of an intra-arterial catheter and drug delivery system and v) a performance status (Eastern Cooperative Oncology Group, ECOG) (23) of level 0–1.

### Treatment regimen of IFN/5-FU combination chemotherapy and follow-up after surgery

After obtaining informed written consent, each patient was treated with subcutaneous administration of IFN-α (OIF, Otsuka Pharmaceutical Co., Tokyo) and an intra-arterial infusion of 5-FU (Kyowa Hakko Co., Tokyo). IFN-α [5×10^6^ U (5 MU)] was administered on Days 1, 3 and 5 of every week (9–11). Continuous infusion chemotherapy (5-FU, 300 mg/mm^3^/day) through the proper hepatic artery was applied 5 days/week for 2 weeks via a catheter connected to a subcutaneously implanted drug delivery system. All anti-cancer therapies were discontinued when adverse effects reached level 2 according to the ECOG classification (23). In addition to serum chemistry, tumor markers such as α-fetoprotein (AFP) and protein induced by vitamin K antagonist or absence (PIVKA-II) were measured at least once every one month. An abdominal CT scan or dynamic magnetic resonance imaging (MRI) was also performed at least once every 3 months following surgery.

### Statistical analysis

Survival curves were constructed using the Kaplan-Meier method (24). Survival curves were compared using the log-rank test. The features of HCC, biochemistry, ICGR-15, and virus status were compared using the Mann-Whitney test. The level of tumor markers (AFP and PIVKA-II) was compared by the Wilcoxon matched-pair test. Statistical significance was interpreted as P<0.05.

## Results

### Features of the preoperative hepatic function, hepatocellular carcinoma and surgery

The features of the preoperative hepatic function are shown in Table I. There was no significant difference between the IFN/5-FU adjuvant and non-IFN/5-FU adjuvant groups in terms of the preoperative hepatic function: serum albumin (g/dl), prothrombin time (PT, %), hepaplastin test (HPT, %), indocyanine green retention rate at 15 min (ICGR-15, %). No difference was also demonstrated in terms of tumor stage, surgical procedure, including AFP (ng/ml) and PIVKA-II (mAU/ml) (Table I).

### Clinical effects, disease-free and overall survival

Concerning the enrolled 50 patients, none developed any major complications. The 30 IFN/5-FU patients started the postoperative adjuvant therapy 3–5 weeks after surgery and completed 3 cycles of treatment. In addition, the QOL of patients in this study was excellent, as this adjuvant therapy was performed at outpatient clinics; no hospital admission was necessary. The patients were able to maintain their social life while receiving IFN/5-FU adjuvant therapy.

The follow-up period of the present study was from 3 to 109 months (mean 24 months). The difference in follow-up was significant compared to the prior study (20). Results for the IFN/5-FU adjuvant treatment group were as follows: disease-free survival (n=9) (15–109 months), survival with recurrence (n=6) (30–92 months), cancer death (n=9) (14–60 months), death from other causes but no recurrence (n=5) (13–87 months) and death from other causes with recurrence (n=1, 22 months). The summary of these results for each case is shown in Table IIA.

In the group that received no adjuvant IFN/5-FU therapy, 14 of 20 patients died of recurrent cancer within 1 year; almost all patients (17 of 20) within 3 years. All patients developed recurrences in the residual liver, 4 also had lung metastasis, and one had lung and lymph node metastases. Recurrence was identified within 1 year after hepatic resection in 16 of the 20 patients. These clinical results are summarized in Table IIB.

**Figure 1:**
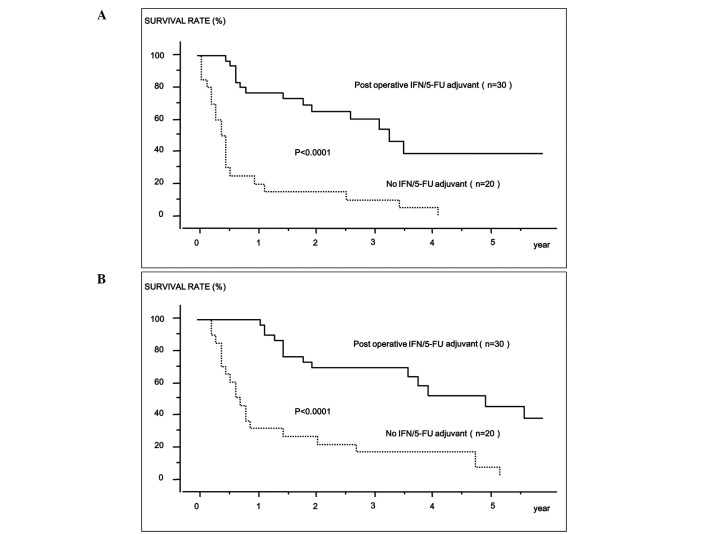
(A) Disease-free survival rates of patients grouped according to whether they received IFN/5-FU combined chemotherapy or not as a postoperative adjuvant following hepatic resection. A statistically significant difference in survival was noted (P<0.0001). (B) Overall survival rates of patients grouped according to whether they received IFN/5-FU combined chemotherapy or not as a postoperative adjuvant following hepatic resection. A statistically significant difference in survival was noted (P=0.0001).

With respect to survival, the 1-, 3- and 5-year disease-free survival rates were 77, 60 and 39% for patients who received IFN/5-FU combination therapy (n=30); 20, 10 and 0%, respectively, for the historical controls (n=20) (Fig. 1A). In addition, the 1-, 3- and 5-year overall survival rates were 100, 69 and 44% for patients who received IFN/5-FU combination therapy (n=30); 30, 15 and 5% for the historical controls (n=20) (Fig. 1B). There was a significant difference in disease-free and overall survival rates between these two groups (disease-free, P<0.0001; overall, P<0.0001) (Fig. 1).

### Adverse effects

No leukopenia, thrombocytopenia, or myelo-suppression was observed in the 30 patients of the IFN/5-FU group. Other adverse effects were, in general, clinically manageable. Fever was commonly observed but was easily controlled by non-steroidal anti-inflammatory drugs prior to IFN injection. No depression due to IFN administration was observed in any of the 30 patients.

## Discussion

The present study is the extended examination of our previous report (20) concerning the clinical outcome of a combination therapy of IFN-α and 5-FU as a postoperative adjuvant therapy for resectable HCC with PVTT. Our results showed that this treatment regimen markedly decreased the incidence of recurrence in the residual liver and significantly prolonged the disease-free and overall survival periods compared with historical controls. Amazingly, the 1-year overall survival rate was 100% in the IFN/5-FU treatment group. This result concerning the survival benefit for resectable far advanced HCC with PVTT was much better than the Japan survey for HCC patients (25).

Development of tumor thrombi in a major branch or main trunk of the portal vein is a frequent terminal feature of HCC, either with primary or recurrent tumors. The prognosis of such patients is extremely poor, and survival is limited to a few months after diagnosis (26–31). For these advanced HCCs, conventional therapies like transcatheter arterial embolization (TAE) and radiofrequency ablation (RFA) are not indicated due to lack of efficacy and associated complications (30–32). Liver transplantation is a contra-indication for such far advanced HCC with PVTT cases (33). To date, several reports have mentioned the feasibility of hepatic resection for patients with PVTT, however the outcome is unsatisfactory (28,29,34,35), except for limited cases with PVTT located in the segmental or sectoral branches (36). Based on this finding, in the absence of effective preoperative and/or postoperative adjuvant therapy, hepatic resection should not be offered in cases with PVTT in the main trunk or first branch. Several approaches have been attempted to improve the surgical results, including radiotherapy and TAE (37–40). Compared with these reports, our clinical outcome using IFN/5-FU combined therapy as a postoperative adjuvant was excellent and highly satisfactory, in terms of survival and long-term outcome.

In regards to recurrence, extrahepatic metastases often occurred following surgery even after IFN/5-FU adjuvant treatment. The combination of IFN/5-FU is not effective against extrahepatic metastases. This is understandable as 5-FU, administered into the hepatic artery, does not reach extrahepatic tissues at a high concentration. In such a situation, systemic administration of 5-FU or related agents may be effective against extrahepatic lesions in combination with IFN-α (41). This possibility is highly interesting since the implantation of a dwelling catheter is one of the demerits of the present combination therapy (9–11). Recently, several molecular-targeting agents have been developed and applied for HCC treatment (42–44). Particularly, sorafenib is the first agent leading to improved overall survival for patients with advanced HCC, as revealed in a phase III clinical trial (10). These molecular-targeting agents are a very effective therapeutic modality, which exhibited a different mechanism of anti-tumor effect compared to IFN/5-FU combination as an cytotoxic medicine. Based on this evidence, mutual interaction and shared roles may be extremely important for the progression of treatment for intractable advanced HCC. We previously reported that PTK/ZK, a type of molecular-targeting medicine, enhanced the anti-tumor effect of IFN/5-FU *in vitro*(45).

Myelosuppressive adverse effects frequently occur in patients with HCC. This is not only because thrombocytopenia and/or leukopenia are often present prior to the initiation of anti-cancer therapy, but because treatment often has to be discontinued due to these side effects. Another advantage of our combination therapy was the markedly low incidence of myelosuppressive side effects; no patient developed leukopenia in the present study (data not shown). In addition, the QOL of patients in the present study was excellent, as IFN/5-FU adjuvant therapy was performed at outpatient clinics. No hospital admission was necessary for the administration of the IFN injection combined with the intra-arterial perfusion chemotherapy. The patients were able to maintain their social life while receiving IFN/5-FU adjuvant therapy. Moreover, they had no symptoms related to liver dysfunction.

In conclusion, this study indicated that combination chemotherapy with subcutaneous IFN-α and intra-arterial 5-FU is a promising strategy for resectable advanced HCC with PVTT in the main trunk or first branch, as a postoperative adjuvant therapy following surgery.

## Figures and Tables

**Table I:** Demographics of the IFN/5-FU and non-IFN/5-FU adjuvant groups. A, IFN/5-FU adjuvant group (n=30)

A, IFN/5-FU adjuvant group (n=30)														
Case	Age	Gender	T	M	N	Vp	Stage	Surgery	Alb	PT/HPT	ICGR-15	AFP	PIVKA-II	Virus
1	47	M	4	0	0	4	4A	Left lobectomy	4.5	81/91	4	11,400	7,900	B
2	69	M	4	0	0	3	4A	Extended anterior segmentectomy	3.7	86/85	21	768	14,784	C
3	54	M	4	0	1	4	4A	Right lobectomy	3.5	64/105	16	28	1,847	B+C
4	47	M	4	0	0	4	4A	Extended right lobectomy	3.4	74/67	26	27	2,067	B+C
5	60	M	4	0	0	3	4A	Extended posterior segmentectomy	3.9	71/69	16	<5	<40	B
6	80	M	4	0	0	4	4A	Left lobectomy	4.0	74/66	26	19	1,568	B+C
7	34	M	4	0	0	4	4A	Extended left lobectomy	3.9	90/89	4	456	1,153	B
8	66	M	4	0	0	3	4A	Extended medial segmentectomy	3.3	75/87	15	5	298	C
9	54	M	4	0	0	4	4A	Right lobectomy	4.5	77/62	14	8,700	353,617	B
10	54	M	4	0	0	4	4A	Right lobectomy	3.7	65/85	21	32,930	<40	B+C
11	69	M	4	0	0	4	4A	Right lobectomy and pancreato-duodenectomy	4.1	90/93	17	7,473	205	B+C
12	54	M	4	0	0	4	4A	Left lobectomy	3.8	82/78	17	680	<40	C
13	56	F	4	0	0	4	4A	Left lobectomy	3.6	71/63	19	13,260	1,039	C
14	62	M	4	0	0	4	4A	Right lobectomy	3.6	63/73	18	23,500	476	B+C
15	58	M	4	0	0	4	4A	Right lobectomy	3.8	85/87	16	6,500	1,200	C
16	63	M	4	0	0	4	4A	Right lobectomy	3.2	93/97	29	390,000	40,775	C
17	58	M	4	0	0	4	4A	Extended left lobectomy	4.2	93/95	6	6,840	51,265	B
18	67	M	4	0	0	3	4A	Extended left lobectomy	4.4	76/81	15	27	48	C
19	61	M	4	0	0	4	4A	Extended left lobectomy	4.0	74/76	21	34	<40	C
20	67	M	4	0	0	4	4A	Right lobectomy	4.1	63/72	8	2,461	32,742	B
21	63	M	4	0	0	4	4A	Right lobectomy and partial resection of lung	3.4	68/85	17	6,325	<40	B
22	58	M	4	0	0	3	4A	Extended posterior segmentectomy	4.4	68/61	12	7	76	B
23	73	M	4	0	0	4	4A	Right lobectomy	3.9	72/71	26	23	<40	B+C
24	56	M	4	0	0	4	4A	Extended left lobectomy	3.5	64/54	24	5	<40	B
25	66	M	4	0	0	4	4A	Left lobectomy	3.5	57/85	20	19,735	97	B
26	58	M	4	0	0	4	4A	Right lobectomy	4.2	78/79	14	486	88	C
27	70	M	4	0	0	4	4A	Left lobectomy	4.3	68/57	7	56,479	282	C
28	62	M	4	0	0	4	4A	Right lobectomy	3.9	77/76	13	89	263	C
29	55	M	4	0	0	3	4A	Extended anterior segmentectomy	4.3	70/72	12	847	1,174	B
30	61	M	4	0	0	4	4A	Extended right lobectomy	3.5	79/83	13	376	2,453	B+C

B, The non-IFN/5FU adjuvant group (n=20)														
Case	Age	Gender	T	M	N	Vp	Stage	Surgery	Alb	PT/HPT	ICGR-15	AFP	PIVKA-II	Virus
31	72	M	4	0	1	3	4A	Right lobectomy	4.3	ND/89	15	10,876	-	-
32	56	M	4	0	0	3	4A	Right lobectomy	3.2	ND/74	23	377	-	-
33	42	M	4	0	0	4	4A	Right lobectomy	4.1	ND/82	14	67	-	B
34	65	M	4	0	0	4	4A	Extended left lobectomy	4.0	ND/133	6	5	-	B
35	58	M	4	0	0	3	4A	Right lobectomy	3.7	ND/72	16	227	-	-
36	61	M	4	0	0	4	4A	Right lobectomy	3.7	ND/75	80	10,256	-	-
37	62	M	4	0	0	3	4A	Leteral segmentectomy	3.8	ND/73	19	105,360	-	-
38	34	M	4	0	0	3	4A	Right lobectomy	2.9	61/59	6	10,332	10,240	B
39	56	M	4	0	0	3	4A	Left lobectomy	3.1	76/52	11	75	1,450	B
40	48	M	4	0	0	4	4A	Right lobectomy	2.8	58/57	14	9	62.5	B
41	54	M	4	0	1	4	4A	Extended right lobectomy	3.0	85/93	-	1,500	21,300	-
42	58	M	4	0	0	4	4A	Right lobectomy	4.2	84/72	19	2,208	62.5	B
43	68	M	4	0	0	3	4A	Extended posterior segmentectomy	4.2	62/65	14	144	6,846	C
44	69	F	4	0	0	4	4A	Right lobectomy	3.7	97/97	7	2,900	571	C
45	67	M	4	0	0	3	4A	Extended posterior segmentectomy	2.9	67/60	35	4,733	18,625	C
46	41	M	4	0	0	3	4A	Extended left lobectomy	3.4	ND/64	13	5	-	B
47	58	M	4	0	0	4	4A	Right lobectomy	4.1	ND/84	19	30,646	-	-
48	63	M	4	0	0	3	4A	Extended posterior segmentectomy	3.4	72/84	15	21	62.5	C
49	76	M	4	0	0	4	4A	Right lobectomy	3.5	85/78	18	16	224	C
50	74	M	4	0	0	4	4A	Right lobectomy	3.1	58/57	14	11	2,098	B+C

TNM stage and the grade of portal vein thrombus were classified according to the 5th edition of the General Rules of the Clinical and Pathological Study of Primary Liver Cancer by the Liver Cancer Study Group of Japan. Alb, serum albumin (g/dl); PT, prothrombin time (%); HPT, hepaplastin test (%); ICGR-15, indocyanine green retention rate at 15 min (%); AFP, α-fetoprotein (ng/ml); PIVKA-II, protein induced by vitamin K antagonist or absence (mAU/ml); ND, not done.

**Table II:** Prognosis and pathological findings of the IFN/5-FU and non-IFN/5-FU adjuvant groups.

A, IFN/5-FU adjuvant group (n=30)								
Case	Recurrence	Recurrent site	Disease-free period	Survival period	Prognosis	Cause of death	Histology of cancer	Non-cancer
1	-	-	68	68	Died	HBV, Lz, Liver failure^a^	EdIII(por)	B′^−^
2	+	Liver	18	60	Died	Cancer	EdII(mod)	CAH^+^
3	-	-	109	109	Alive	-	EdII(mod)	B^−^
4	-	-	87	87	Died	Brain bleeding^a^	EdIII(por)	CAH^+^
5	+	Liver, lung	40	48	Died	Cancer	EdIII(por)	B′^−^
6	+	Liver, lung, adrenal	38	46	Died	Cancer	EdIII(por)	B′^−^
7	+	Liver, lung	7	18	Died	Cancer	EdIII(por)	B′^−^
8	+	Liver	8	22	Died	Cardiac failure^b^	EdII(mod)	B′^−^
9	-	Lung	28	92	Alive	-	EdIII(por)	B′^−^
10	+	Lymph node, liver	6	24	Died	Cancer	EdIII(por)	B^−^
11	+	Liver	43	75	Alive	-	EdIII(por)	B′^−^
12	-	-	44	44	Died	Cardiac failure^a^	EdIII(por)	B′^−^
13	+	Liver	32	73	Alive	-	EdII(mod)	B^−^
14	+	Lung, liver	8	14	Died	Cancer	EdIII(por)	B′^−^
15	-	-	13	13	Died	Brain infarction^a^	EdII(mod)	CAH^+^
16	-	-	18	18	Died	Esophageal varix^a^	EdIV(por)	B^+^
17	+	Liver	9	18	Died	Cancer	EdIII(por)	chr.glissonitis
18	+	Liver	24	51	Alive	-	EdII(mod)	CIH
19	-	-	46	46	Alive	-	EdIII(por)	CAH^+^
20	+	Lung, liver	8	14	Died	-	EdIII(por)	Liver fibrosis
21	-	-	38	41	Alive	-	EdIV(por)	B′^−^
22	-	-	38	38	Alive	-	EdII(mod)	B′^+^
23	-	-	37	37	Alive	-	EdIII(por)	B′^−^
24	-	-	36	36	Alive	-	EdIII(por)	B′^−^
25	-	-	31	31	Alive	-	EdII(mod)	CIH
26	-	-	30	30	Alive	-	EdIII(por)	B^−^
27	+	Lymph node	22	30	Alive	-	EdIII(por)	B′^−^
28	-	-	24	24	Alive	-	EdIII(por)	B^−^
29	+	Liver, lung	10	16	Died	Cancer	EdIII(por)	CIH
30	-	-	15	15	Alive	-	EdIII(por)	CAH^+^

B, The non-IFN/5-FU adjuvant group (n=20)								
Case	Recurrence	Recurrent site	Disease-free period	Survival period	Prognosis	Cause of death	Histology cancer	Non-cancer
31	+	Liver	50	58	Died	Cancer	EdIII(por)	CAH
32	+	Liver, lung	1	3	Died	Cancer	EdIII(por)	B′^−^
33	+	Liver	1	5	Died	Cancer	EdIII(por)	B′^−^
34	+	Liver	12	18	Died	Cancer	EdIII(por)	Unknown
35	+	Liver	5	5	Died	Cancer	EdII(mod)	BA′^+^
36	+	Liver	42	63	Died	Cancer	EdIII(por)	B^+^
37	+	Liver	4	33	Died	Cancer	EdIII(por)	B^+^
38	+	Liver	5	10	Died	Cancer	EdIII(por)	CIH^−^
39	+	Liver	3	5	Died	Cancer	EdIII(por)	Glissonitis,se
40	+	Liver	2	4	Died	Cancer	EdIV(undifferentiated)	Glissonitis
41	+	Liver	1	3	Died	Cancer	EdIV(undifferentiated)	Chr.glissonitis
42	+	Liver	31	58	Died	Cancer	EdIII(por)	B^−^
43	+	Liver, lung, lumph node	6	6	Died	Cancer	EdIII(por)	Chr.glissonitis
44	+	Liver, lung	3	8	Died	Cancer	EdIII(por)	CIH
45	+	Liver	4	7	Died	Cancer	EdIII(por)	B^−^
46	+	Liver	6	9	Died	Cancer	EdII(mod)	B′^−^∼A′^++^
47	+	Liver, lung	6	8	Died	Cancer	EdIII(por)	CIH
48	+	Liver, lung	14	25	Died	Cancer	EdIII(por)	B^+^
49	+	Liver	6	10	Died	Cancer	EdIII(por)	CIH
50	+	Liver	7	11	Died	Cancer	EdIII(por)	B′^+^

a Death from other causes but no recurrence;

b Although the recurrent lesion completely disappeared after the re-treatment with IFN/5-FU combined chemotherapy (CR), the patient died suddenly due to cardiac failure of ischemic disease. Disease-free and survival periods are expressed in months.

## Abbreviations:

HCC: hepatocellular carcinoma
IFN: interferon
AFP: α-fetoprotein
PIVKA-II: protein induced by vitamin K antagonist or absence
PVTT: portal vein tumor thrombosis
5-FU: 5-fluorouracil
TAE: transcatheter arterial embolization
RFA: radiofrequency ablation
